# An Unexpected Diagnostic Twist in an Elderly Patient: No Heart Failure With Preserved Ejection Fraction

**DOI:** 10.7759/cureus.55971

**Published:** 2024-03-11

**Authors:** Samuel Vysočanský, Milan Luknár, Peter Lesný, Jana Poláková-Mištinová, Eva Goncalvesová

**Affiliations:** 1 Department of Cardiology, Heart Failure and Heart Transplant Unit, Comenius University Faculty of Medicine, Bratislava, SVK; 2 Department of Radiology, Cardiac Magnetic Resonance Unit, Comenius University Faculty of Medicine, Bratislava, SVK; 3 Slovak Medical Faculty, National Institute of Cardiovascular Diseases, Bratislava, SVK

**Keywords:** adult congenital heart disease, right heart catheterization, partial anomalous pulmonary venous return, pulmonary hypertension, heart failure with preserved ejection fraction

## Abstract

Heart failure with preserved ejection fraction (HFpEF) is considered to be the dominant cause of dyspnea and pulmonary hypertension (PH) in elderly patients with preserved left ventricular systolic function and cardiovascular comorbidities. However, it is important to keep in mind that left ventricular diastolic dysfunction is not the only possible cause of PH in cases of late-onset clinical manifestation. A multiparametric approach is essential for accurate diagnosis and therapeutic decision-making. A 74-year-old patient was admitted due to progressive dyspnea and suspicion of PH. Given the patient's risk profile, HFpEF and concomitant post-capillary PH were anticipated. Despite negative findings on CT angiography and transesophageal echocardiography, right heart catheterization was performed, revealing discrepant oxygen saturations in the superior vena cava and right atrium. A partial anomalous pulmonary venous return and an atrial septal defect were identified through cardiac magnetic resonance imaging.

## Introduction

Partial anomalous pulmonary venous return (PAPVR) is defined as one or more, but not all, pulmonary veins draining blood directly into the right atrium or indirectly through a systemic vein. The overall incidence is estimated to be 0.7% of the population based on the autopsy series [1]. A recent retrospective study using chest CT indicated a condition prevalence of 0.1% [2]. Studies based on the pediatric population suggest that PAPVR involving the right pulmonary vein might be associated with a concurrent sinus venosus atrial septal defect (ASD) [3].

PAPVR and ASD are congenital heart defects that cause pathologic left-to-right shunting. Patients with either of these defects may be asymptomatic; however, they can rarely present with clinical symptoms at an advanced age. The clinical presentation depends on the degree of left-to-right shunting, which may be influenced by other cardiovascular comorbidities in elderly patients. Any condition negatively affecting the left ventricle compliance or causing an elevation of the left atrium pressure increases the left-to-right shunt fraction. On the other hand, a reduction in right ventricle compliance may decrease left-to-right shunting or eventually cause shunt reversal. From a pathophysiological standpoint, left-to-right shunting leads to an increase in recirculating blood flow, primarily affecting the right heart chambers. There are also significant consequences regarding the pulmonary circulation. Prolonged pathologic shunting and volume overload lead to the development of PH. Fixed changes in the precapillary pulmonary vasculature are characteristic features of pulmonary vascular disease. Increasing right heart afterload may lead to shunt reversal (change to the right-to-left shunt), also known as Eisenmenger´s syndrome [4].

## Case presentation

**Figure 1:**
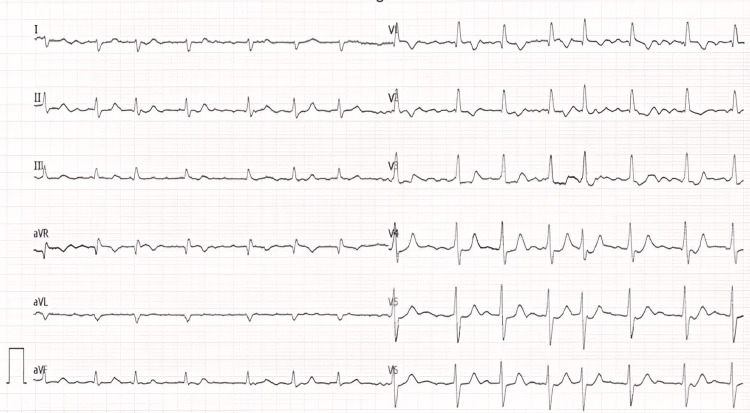
AF, right heart axis deviation, incomplete right bundle branch block, negative T waves in right precordial leads on the initial electrocardiogram AF: atrial fibrillation

A 74-year-old patient with a history of systemic hypertension and new-onset atrial fibrillation (AF) was referred for a diagnostic check-up because of dyspnea and intermittent chest tightness. Blood pressure during admission was 181/110 mm Hg. There were discrete signs of systemic venous congestion: jugular vein distension, hepatomegaly, and perimalleolar edema. Accentuated second heart sounds above the pulmonary valve and a systolic murmur in the right parasternal base were present. AF with a heart rate of 85 bpm, right heart axis deviation, incomplete right bundle branch block, and negative T waves in the right heart precordial leads were present on the initial electrocardiogram (Figure 1). Natriuretic peptides were significantly elevated (NT-proBNP = 4086 ng/L), and there was also an elevation of serum bilirubin (57.1 umol/L).

**Figure 2:**
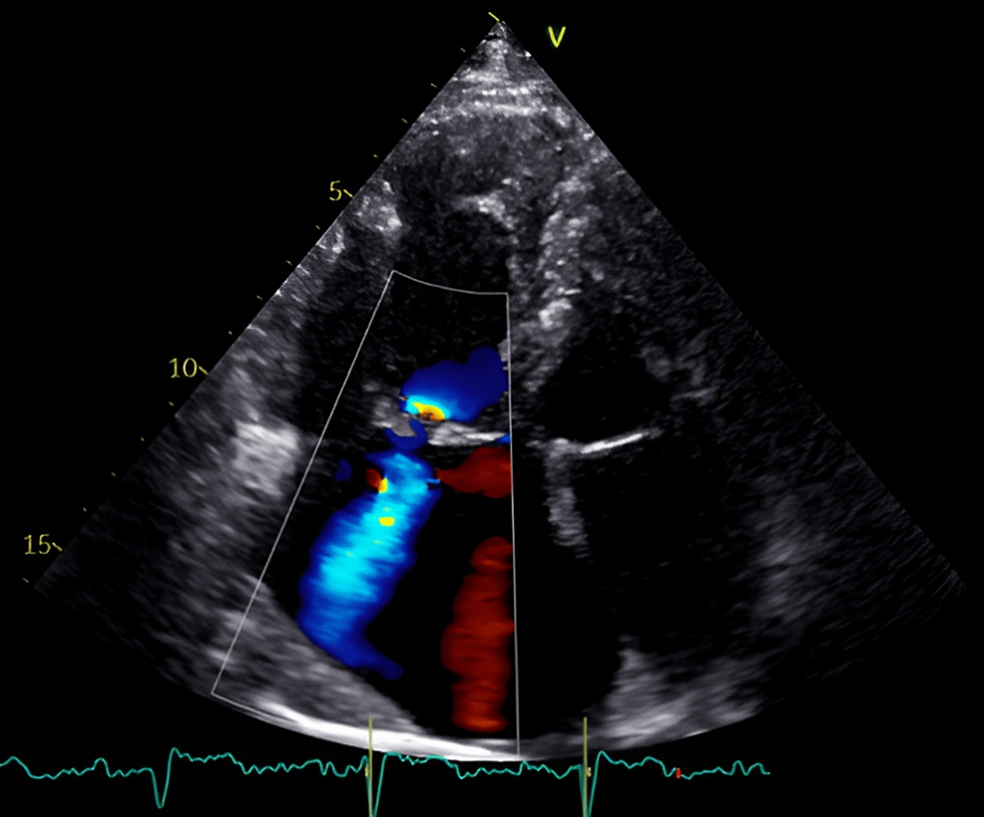
TTE (apical four-chamber view) showing moderate tricuspid regurgitation, significant dilatation of right heart chambers and left atrium TTE: transthoracic echocardiography

Echocardiography revealed a normal-sized left ventricle with preserved ejection fraction (left ventricular ejection fraction 65%), a flattened interventricular septum leading to a D-shaped left ventricle, significant dilatation of the right ventricle, and both atria. Moderate mitral regurgitation was present. Regarding the right heart abnormalities, we observed moderate tricuspid regurgitation with an estimated sPAP of 70 mm Hg (Figure 2). Probable pulmonary hypertension (PH) increased left atrial indexed volume (55 mL/m2), and the assessment of mitral annulus velocity (average e´ 6 cm/s, E/e´ ratio 20) by Doppler tissue imaging was suggestive of elevated left ventricle filling pressure. Consistent with these findings, there was also the presence of B-lines on lung ultrasonography, indicative of subclinical lung congestion.

**Figure 3:**
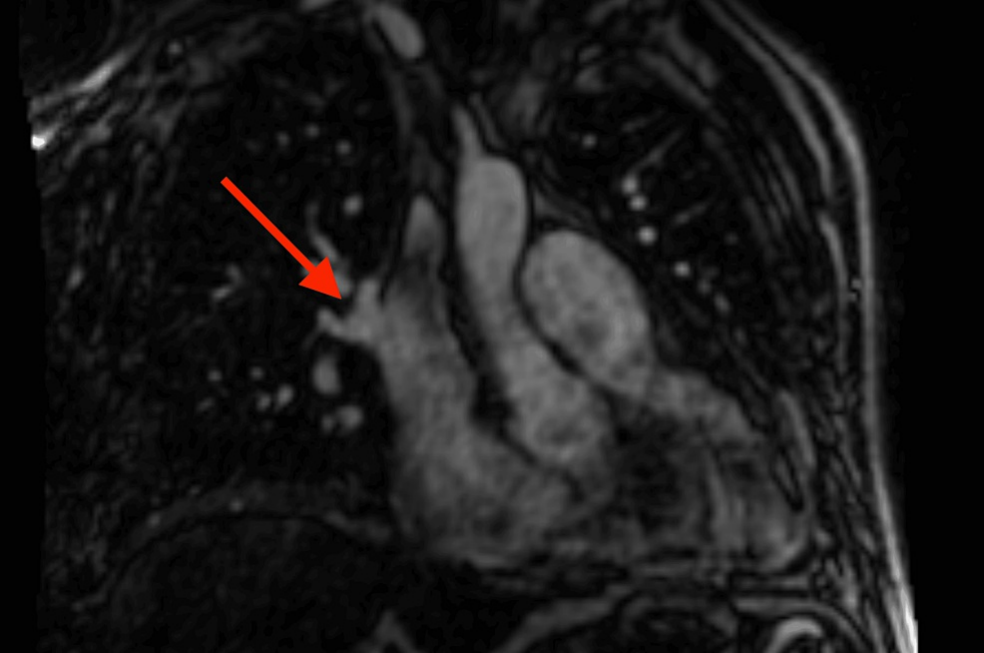
PAPVR: right pulmonary veins draining blood to the SVC (red arrow) on the CMR PAPVR: partial anomalous pulmonary venous return, SVC: superior vena cava, CMR: cardiac magnetic resonance

**Figure 4:**
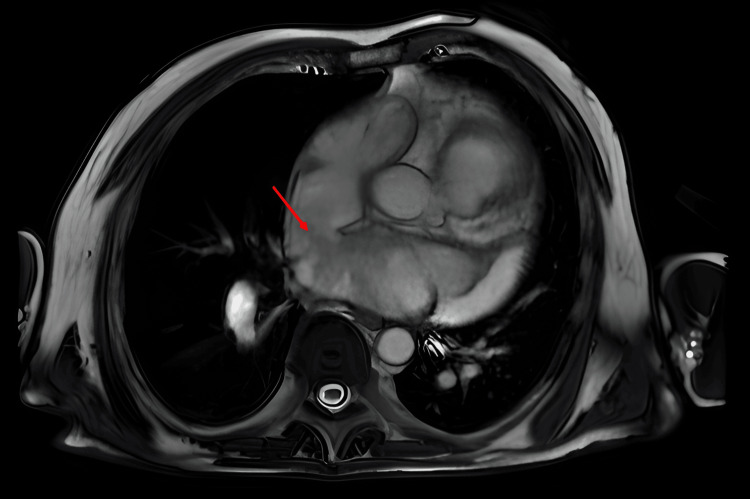
ASD: sinus venosus superior (red arrow) with significant left-to-right shunting on the CMR ASD: atrial septal defect, CMR: cardiac magnetic resonance

Based on the patient profile, comorbidities, and echocardiographic findings, we assumed that the patient had heart failure with preserved ejection fraction (HFpEF) with consequent post-capillary PH. Another possible cause of clinical manifestation could be recently defined pulmonary arterial hypertension (PAH) with cardiopulmonary comorbidities. Both available score-based algorithms, H2FPEF (7 points, 99.1% probability of HFpEF) and HFA-PEFF (6 points), indicated a high probability of HFpEF. Considering the history of chest tightness, we performed selective coronary angiography with concomitant measurement of left ventricle end-diastolic pressure after reaching euvolemic status. The coronary angiogram was normal, as was the left ventricle filling pressure (10 mm Hg), which challenged our initial hypothesis of post-capillary PH. Causes of pre-capillary PH seemed to be excluded as the chest CT scans didn´t show any lung tissue abnormalities or signs of chronic thromboembolic disease. Anomalous pulmonary venous return was not identified. Transesophageal echocardiography was performed by an experienced clinician, showing a small patent foramen ovale; any significant ASD was ruled out. Pulmonary function tests did not reveal an obstructive or restrictive pulmonary disease. We performed right heart catheterization (RHC) to evaluate central hemodynamics and classify PH. RHC found mild pre-capillary PH (Table 1). There was a significant difference in oxygen saturation between the superior vena cava (SVC) (71%), and the right atrium (RA) (87%), which led to a suspicion of a shunt between the pulmonary and systemic circulation despite negative imaging studies so far. We used the thermodilution method for the estimation of cardiac output. Finally, cardiac magnetic resonance (CMR) revealed PAPVR with right pulmonary veins draining blood to SVC (Figure 3) and a sinus venosus superior ASD with hemodynamically significant left-to-right shunting (Qp:Qs - 3.2) (Figure 4). CMR results led to the diagnosis of PAH associated with adult congenital heart disease.

**Table 1:** Complete set of hemodynamic parameters obtained during RHC CO: cardiac output, CI: cardiac index, d: diastolic, m: mean, PAP: pulmonary artery pressure, PAWP: pulmonary artery wedge pressure, PVR: pulmonary vascular resistance, s: systolic, WU: wood unit, PH: pulmonary hypertension, RHC: right heart catheterization * According to the 2022 ESC/ERS Guidelines for the diagnosis and treatment of PH

	Patient	Normal value*
RAP (mm Hg)	8	2-6
PAP s/d/m (mm Hg)	50/13/26	15-30/4-12/8-20
PAWP (mm Hg)	11	≤15
CO (L/min)	6.8	4-8
CI (L/min/m^2^)	3.5	2.5-4.0
PVR (WU)	2.2	0.3-2.0

The patient´s case was discussed with a multidisciplinary heart team (cardiologist, cardiac surgeon, cardiac MRI specialist, and anesthesiologist) in order to consider the possibility of surgical repair of PAPVR and ASD closure. The European System for Cardiac Operative Risk Evaluation II was used for risk estimation. Considering renal insufficiency, mild PH, and the New York Heart Association class, the predicted risk of in-hospital mortality after major cardiac surgery reached 5%. The patient preferred medical therapy, seeking partial symptom relief, and avoiding the surgery as much as possible. Considering all relevant and decisive aspects, he was contraindicated for any intervention. The conservative approach included sinus rhythm restoration by electric cardioversion and the initiation of PAH-specific monotherapy with tadalafil. Combined antihypertensive therapy and warfarin were recommended as before, and a small dose of furosemide was added to the therapy.

The patient was reevaluated at the outpatient clinic three months after discharge. His functional capacity has markedly improved. There was a recurrence of AF with a heart rate of 100 bpm. An echocardiographic study showed a regression of tricuspid regurgitation severity with a decrease in estimated systolic pulmonary artery pressure by 20 mm Hg. The diameters of the right ventricle were almost identical. We decided to aim for a heart rate control strategy because of an early AF recurrence by adding metoprolol to the therapy. Specific PAH therapy with tadalafil is being continued.

## Discussion

PAPVR is a rare congenital heart defect with variable clinical manifestations. Occasionally, it may be described as an incidental finding in asymptomatic adult patients. The most frequent symptoms caused by PAPVR are fatigue, dyspnea, or recurrent pneumonia. The decisive factor that contributes to the clinical presentation is the degree of left-to-right shunting. Any condition affecting the compliance of the left ventricle, leading to the elevation of left atrial pressure, increases left-to-right shunting, which might be the reason for late-onset symptoms in elderly patients with cardiovascular comorbidities.

Diagnosis of PAPVR is challenging, especially in elderly patients presenting with other cardiovascular comorbidities. A combination of non-invasive imaging studies and invasive diagnostic methods is necessary. Diagnosis is rarely based on transthoracic echocardiography (TTE) due to poor visualization of the superior posterior atrial septum. Contrast echocardiography with intravenous agitated saline was not performed in our patient, but it may enhance the sensitivity of TTE in diagnosing ASD via visualization of the bubbles traveling across the defect. In cases of superior ASD with an overriding superior vena cava, bubbles can appear nearly simultaneously in both atria due to a small amount of right-to-left shunting, although the predominant hemodynamic consequence of the defect is left-to-right shunting [5]. TEE is indicated in all cases of suspected congenital heart disease with nondiagnostic or inadequate TTE images. TEE is usually highly sensitive and specific in the detection of PAPVR and ASD using standard projections; missing these pathologies is rare but possible. CMR is able to visualize shunt anatomy, quantify shunt fraction, and provide information regarding the right ventricle function [6]. The only disadvantage regarding the CMR is its lower availability compared to the diagnostic methods mentioned before. CMR presents a better diagnostic option for patients with inferior sinus venosus defects, which might be missed by the TEE. It is important to keep in mind that some cardiovascular institutes have better access to invasive diagnostic methods, which might be the reason for RHC being performed in the early stages of the diagnostic process. RHC remains the gold standard for assessing pulmonary hemodynamics, also contributing to the diagnosis by revealing differences in oxygen saturation. It might be helpful in cases of inconclusive echocardiography studies, as we showed, and is crucial for making the right therapeutic decision in patients with PH and PAPVR/ASD. Any abnormal step-up in oxygen saturation should be followed, preferably by CMR or cardiac CT.

Surgical indications and strategies remain controversial considering the lack of robust data, especially in elderly patients. Furthermore, the surgical procedure poses a challenge due to the potential risk of inducing thrombosis in the surgically treated vein. PAPVR/ASD surgical correction is recommended in symptomatic patients with signs of right ventricle overload, recurrent lung infections, and significant left-to-right shunting (Qp:Qs > 1.5) in the absence of severe pulmonary vascular disease (PVR < 5WU) [7,8]. Surgery is not recommended in patients with severe PAH, PVR > 5 WU despite specific therapy, or in patients with Eisenmenger physiology. Good long-term outcomes are feasible when the surgery is performed early, in the absence of elevated pulmonary pressures. In patients with impaired LV function (systolic/diastolic), ASD closure may worsen heart failure. If the risk associated with the surgery outweighs the potential benefit, PAH-specific therapy may be considered. There is limited evidence regarding the conservative treatment of left-to-right shunts. Guidelines refer to the “treat and repair” strategy in patients with severe pulmonary vascular disease as a potential bridge to surgery option, without any preference among pulmonary vasodilators being used in PAH [8]. Improvement in quality of life and symptom relief are the therapeutic targets in these patients, significant hemodynamic improvement is unlikely. The majority of data for specific PAH therapy in patients with ASD and/or PAPVR is supported by small studies that have shown symptomatic and hemodynamic benefits in patients with Eisenmenger's syndrome.

## Conclusions

This case, highlighting late-onset PAH associated with adult congenital heart disease initially resembling HFpEF, underscores the imperative need for a comprehensive diagnostic approach in patients with PH. PAPVR may be easily overlooked or misidentified as other PH causes, particularly in elderly individuals with cardiovascular comorbidities. A multimodal approach, including RHC with multiple-site oxygen saturation assessment, is essential while acknowledging the limitations of each diagnostic method.

The combination of invasive and non-invasive methods is indispensable for guiding therapeutic interventions. Although HFpEF scoring systems serve as valuable diagnostic tools, heightened suspicion for alternative PH causes, particularly in the presence of severe right heart chamber enlargement, is crucial. Typically, PAPVR and/or ASD are suspected on TTE when there is marked right heart dilatation due to the volume overload without an obvious etiology such as significant tricuspid or pulmonic regurgitation. Moreover, impaired left ventricular diastolic function may act as a triggering factor, leading to the clinical manifestation of intra- and extracardiac shunts. A wide range of clinical conditions leading to right heart dilatation underscores the importance of comprehensive diagnostic strategies in managing these patients.

## References

[1] Pendela VS, Tan BE, Chowdhury M, Chow M (2020). Partial anomalous pulmonary venous return presenting in adults: a case series with review of literature. Cureus.

[2] Ho ML, Bhalla S, Bierhals A, Gutierrez F (2009). MDCT of partial anomalous pulmonary venous return (PAPVR) in adults. J Thorac Imaging.

[3] Sung WK, Au V, Rose A (2012). Partial anomalous pulmonary venous return in patients with pulmonary hypertension. J Med Imaging Radiat Oncol.

[4] Chaaban N, Shah H, Joshi A, Kshatriya S (2022). Partial anomalous pulmonary venous return in adults. Cureus.

[5] Qiu JK, Bamira D, Vainrib AF, Latson LA, Halpern DG, Chun A, Saric M (2022). Multimodality imaging of sinus venosus atrial septal defect: a challenging diagnosis in adults. CASE (Phila).

[6] Robinson BL, Kwong RY, Varma PK, Wolfe M, Couper G (2014). Magnetic resonance imaging of complex partial anomalous pulmonary venous return in adults. Circulation.

[7] Humbert M, Kovacs G, Hoeper MM (2022). 2022 ESC/ERS guidelines for the diagnosis and treatment of pulmonary hypertension. Eur Heart J.

[8] Baumgartner H, De Backer J, Babu-Narayan SV (2021). 2020 ESC guidelines for the management of adult congenital heart disease. Eur Heart J.

